# Assessing inter-rater reliability of MRI features in glioma: a multi-radiologist agreement study

**DOI:** 10.1186/s12880-025-01941-5

**Published:** 2025-11-20

**Authors:** Alisa Mohebbi, Saeed Mohammadzadeh, Amir Hessam Zare, Zahra Moradi, Ali Abbasian Ardakani, Afshin Mohammadi

**Affiliations:** 1 Universal Scientific Education and Research Network (USERN), Tehran, Iran; 2 School of Medicine, Tehran University of Medical Sciences, Tehran, Iran; 3 Department of Radiology Technology, School of Allied Medical Sciences, Shahid Beheshti University of Medical Sciences, Tehran, Iran; 4 Department of Radiology, Faculty of Medicine, Urmia University of Medical Science, Urmia, Iran

**Keywords:** Glioma, VASARI features, Inter-rater reliability, Inter-Observer agreement, Brain MRI, Fleiss’ kappa, Gwet’s AC, Brain tumor imaging

## Abstract

**Objective:**

To evaluate the inter-rater reliability of the Visually AcceSAble Rembrandt Images (VASARI) feature set in glioma imaging assessment.

**Materials and methods:**

Three board-certified radiologists independently analyzed 33 adult type glioma cases (20 glioblastomas, seven astrocytomas, five oligodendrogliomas, and one undefined subtype glioma) using 26 VASARI features and three additional non-VASARI features. Inter-rater reliability was assessed using Fleiss’ kappa and Gwet’s AC with conditional confidence intervals. Ordinal weighting was applied to relevant features.

**Results:**

High inter-rater reliability was observed for multifocality, enhancement quality, cyst presence, enhanced tumor crossing of midline, and calvarial remodeling. There was poor agreement for diffusion characteristics, hemorrhage presence, involvement of eloquent brain regions, and T1/FLAIR ratio. Several features, including ependymal invasion and tumor size, showed borderline reliability.

**Conclusion:**

Inter-rater reliability varied across the 29 features evaluated for glioma imaging. Eleven features demonstrated high reliability, and four showed poor agreement. The remaining features exhibited varying levels of reliability, with some falling in a borderline range, indicating areas for potential improvement in standardization and assessment.

**Clinical trial number:**

N/A.

**Supplementary Information:**

The online version contains supplementary material available at 10.1186/s12880-025-01941-5.

## Introduction

Gliomas are the most prevalent primary brain tumors, originating from glial or precursor cells [1]. These tumors encompass a variety of subtype, such as astrocytoma, oligodendroglioma, ependymoma, and glioblastoma, the latter of which is particularly aggressive and associated with a poor prognosis [1, 2]. Precise preoperative assessment of gliomas is crucial for optimal treatment planning and patient management [3].

Magnetic resonance imaging (MRI) is the standard imaging modality for brain tumors, essential for preoperative and postoperative evaluations [4, 5]. Although there is agreement on standardized neuro-oncologic imaging protocols, the reporting of glioma radiophenotypes is still not uniformly standardized [5]. Standardized reporting systems have become increasingly important to enhance the consistency and reproducibility of glioma imaging interpretations [6]. Recent developments in the genetic characterization of gliomas have led to a growing interest in radiogenomics, which is the classification of genetically defined structures according to imaging characteristics. While radiogenomics is often quantitative and relies on large data analysis, visual and qualitative methods are practical and helpful, with visual assessment being the most common approach in clinics [7]. One such system is the Visually AcceSAble Rembrandt Images (VASARI) feature set, developed approximately a decade ago by The Cancer Genome Atlas project of the National Cancer Institute [8]. The VASARI feature set combines various MRI characteristics, including enhancement patterns and tumor location. It was defined by a consensus group based on expert opinion and literature [5, 9]. Since its introduction, the VASARI set has been utilized in numerous studies across diverse contexts, encompassing human radiologic assessments, prediction models, and machine-learning approaches [10, 11]. Such controlled reporting terminology is essential in research settings for identifying reproducible imaging glioma biomarkers and generating data suitable for artificial intelligence approaches [12].

The inter-reader agreement on VASARI features in gliomas has not been extensively studied, representing a substantial clinical knowledge gap. This study aims to address the gap by assessing the inter-rater agreement of different brain MRI features using the VASARI framework. The current study involves analyzing a dataset consisting of brain MRI scans that were assessed by three radiologists using the VASARI feature set, with the objective of identifying areas of consistency and potential discrepancies in glioma imaging interpretation, thereby further enhancing diagnostic accuracy and treatment planning based on selecting the most reproducible MRI features in clinical guidelines.

## Materials and methods

The protocol of the present study was pre-registered on Open Science Framework (OSF) platform, accessible through (https://osf.io/4kht9/) (see Appendix A). REMBRANDT dataset consisting of 33 adult patients who were diagnosed with glioma, sourced from the Thomas Jefferson University (TJU) dataset, was utilized. The data was obtained from the institutional database and was accessed through The Cancer Imaging Archive (TCIA) [13]. Patients’ available consent was secured for this study due to open access and accessible data availability. Handbook of Inter-Rater Reliability [14] has been used as the basis of methodology and analysis in this study.

### Imaging acquisition and segmentation

Pre- and post-operative MRI scans of 33 patients were obtained from the TCIA imaging archive. A board-certified radiologist meticulously annotated all modalities in the dataset, encompassing T1-weighted, T2-weighted, post-contrast T1-weighted (T1-C), T2 Fluid-Attenuated Inversion Recovery (FLAIR) image sequences and, apparent diffusion coefficient (ADC) maps. The segmentation method was utilized to provide consistent anatomical references for radiologists, ensuring that all radiologists evaluated the same anatomical regions when assessing the features. Specifically, the segmentations were used to guide the identification of tumor components such as enhancing tumor, non-enhancing tumor (nCET), necrosis, and edema, ensuring that all radiologists evaluated the same regions when assessing the features. Comprehensive details on imaging acquisition and segmentation protocols are available in the data-processing article by Sayah et al. [15], which elucidates the methodologies of the dataset used in our research. For segmentation, two established algorithms were employed in mentioned study: BraTumIA and GLISTRboost. While BraTumIA proved effective for glioblastoma segmentation, GLISTRboost demonstrated superior performance across all brain tumor subtypes. Therefore, segmentations produced by GLISTRboost were selected for further analysis in Sayah et al. [15]. These segmentations were enhanced through a supplementary manual seeding process using MITK software, ensuring accurate delineation of tumor components.

### Data collection

The MRI data used in this study were accessed through TCIA, specifically from the REMBRANDT collection [13] incorporating each radiologists interpretation. This collection includes pre-surgical MRI scans from glioma patients, which were utilized for evaluating the inter-rater reliability of VASARI features. Three board-certified radiologists evaluated the pre- and post-operative studies independently. The researchers conducted separate measurements of tumors in pre-operative examinations for a set of 26 radiologic features of the tumors, according to the standard definition of VASARI feature set. Besides the VASARI set, three additional identified features, selected for their clinical relevance, were evaluated using both pre- and post-operative imaging. Tumor location was categorized by its epicenter’s location (frontal, temporal, insular, parietal, occipital, brainstem, cerebellum) and side (right, center/bilateral, left). Involvement of eloquent brain regions was assessed (none, speech motor, speech receptive, motor, vision). Enhancement quality was classified as none, mild/minimal, or marked/avid based on post-contrast T1-weighted images. The study quantified proportions of enhancing tumor, non-contrast-enhancing tumor (nCET), necrosis, and edema, each categorized as none (0%), < 5%, 6–33%, 34–67%, 68–95%, > 95%, or all (100%). Tumor morphology was characterized by the presence of cysts (yes or no) and multifocality (multifocal, multicentric, gliomatosis, or none). The T1/FLAIR size ratio was evaluated as expansive, mixed, or infiltrative. Enhancing margins were evaluated for thickness (none, thin, thick/solid) and definition (well-defined, poorly-defined). Non-enhancing margins were assessed as smooth or irregular.

**Fig. 1:**
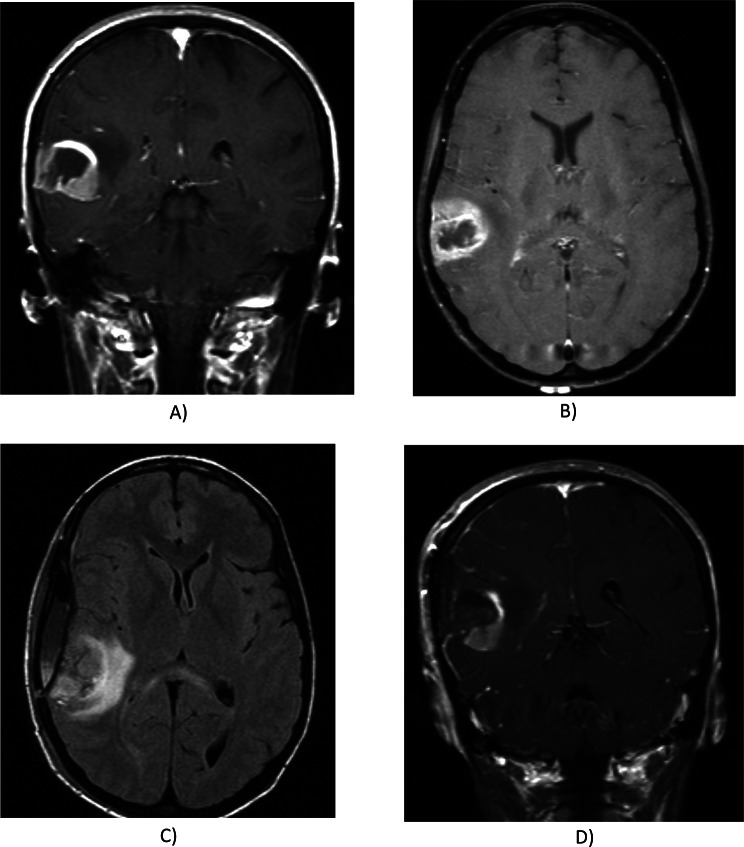
MRI scans of a glioblastoma multiforme: Panels **A** and **B** depict pre-operative images, while panels **C** and **D** show post-operative images. All three radiologists identified a right temporal multifocal mass characterized by smooth thick well-defined enhancing margins, marked enhancing, 6–33% necrosis, cortical and pial involvement, and non-cystic, non-eloquent features without ependymal invasion. No calvarial remodeling or deep white matter involvement, or satellite lesions were noted. 6–33% enhancement was observed by R2, while R1 and R3 reported 34–67% enhancement. The proportion of non-contrast-enhancing tumor (nCET) varied among readers: R1 reported < 5%, R2 6–33%, and R3 none. The size of FLAIR abnormality exceeded pre-contrast T1 in R3’s report, while other readers described equal sizes. Edema proportion was 6–33% according to R1 but was 34–67% in other interpretations. The edema and nCET crosses the midline in R1’s opinion unlike other interpretations while all radiologists agree that enhancing tumor does not cross the midline. The lesion is hemorrhagic in R1’s report unlike other readers. The lesion has diffusion restriction based on R3 while equivocal based on other radiologists’ opinions. Post-operative nCET and enhancing tumor resection was reported none and 34–67% by all radiologists. Lesion dimensions were measured as 3.5 × 2.5 cm by R1 and 4.5 × 3.5 cm by others

**Fig. 2:**
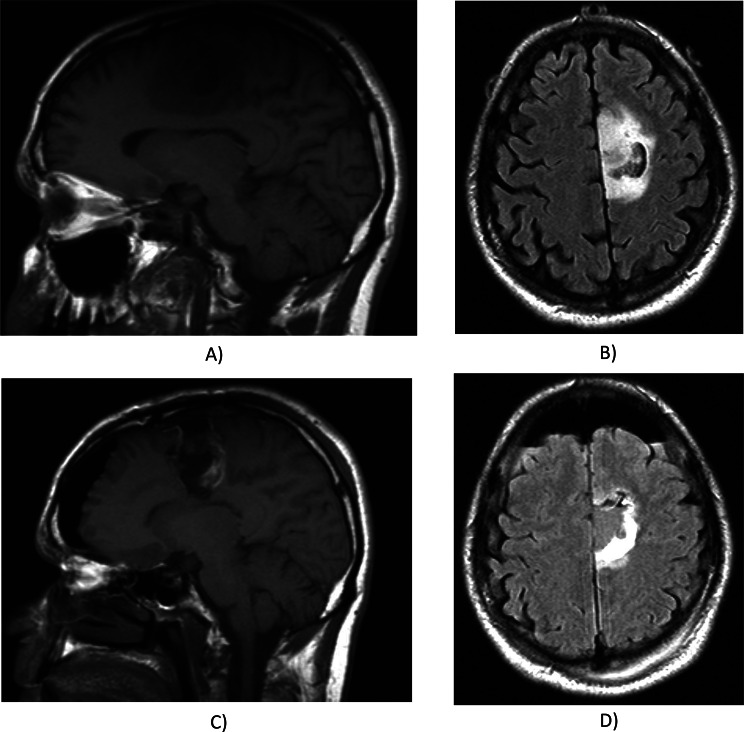
MRI scans of a grade 2 oligodendroglioma: Panels **A** and **B** depict pre-operative images, while panels **C** and **D** show post-operative images. All three radiologists identified a left frontal unifocal mass characterized by smooth margins, cortical involvement, and non-necrotic, non-crossing, non-hemorrhagic, features without pial or ependymal invasion. No diffusion restriction, calvarial involvement, or satellite lesions were noted. R2 noted involvement of eloquent cortex, whereas R1 and R3 reported no such involvement. Mild enhancement (< 5%) was observed by R2, while R1 and R3 reported no enhancement. The proportion of nCET varied among readers: R1 reported 68–95%, R2 > 95%, and R3 100%. Cysts were noted exclusively by R3. The size of FLAIR abnormality exceeded pre-contrast T1 in R2’s report, while other readers described equal sizes. Additionally, R2 identified a thin, poorly-defined enhancing margin absent in other reports. Edema proportion was < 5% according to R1 but was absent in other interpretations. Deep white matter invasion was noted solely by R3. Post-operative nCET resection was reported as > 95% by R3 and 68–95% by others. Lesion dimensions were measured as > 8.0 × 7.5 cm by R1 and 7.5 × 5.5 cm by others

Additional features included hemorrhage presence (yes/no) and diffusion characteristics (facilitated, restricted, or equivocal) based on ADC maps. Invasion patterns were noted based on locations, including pial, ependymal, cortical, and deep white matter involvement (all as yes or no). The study assessed whether tumor components crossed the midline and assessed the presence of satellite lesions. The extent of resection was quantified for enhancing tumor, nCET, and edema using the first postoperative MRI, categorized similarly to tumor proportions. Lesion size was measured as the largest cross-sectional diameter on T2-weighted imaging, with specific size categories ranging from < 0.5 cm to > 8.0 cm in 0.5 cm intervals (scoring from 0 to 18). A comprehensive explanation and details of each variable can be further found on the TCIA website [11]. Also, the clinical examples are provided in Figs. 1 and 2. VASARI features are primarily designed for pre-surgical with our analysis including the 26 core pre-operative VASARI features. However, we included three additional non-VASARI features in our study that require both pre- and post-operative MRI scans. The three features are assessment of the extent of resection of enhancing tumors (F26), nCETs (F27), and vasogenic edema (F28), respectively. These features need post-operative MRI acquisition, so they may not be evaluated if this option is unavailable in clinical centers.

### Methodology and statistical analysis

Fleiss’ kappa and Gwet’s agreement coefficient (AC) were employed to assess inter-rater reliability for each feature, utilizing STATA version 17.0 and MedCalc 22.0. The magnitude of Gwet’s AC was prioritized over Fleiss’ kappa as it provides a more robust estimate of agreement, especially in the case of dealing with unbalanced rating distributions or a high prevalence of a particular category [14]. A cut-off value of 0.6 was defined to assess the acceptability of the features’ reliability. Also, the agreement was interpreted as poor when < 0, slight when 0-0.2, fair when 0.2–0.4, moderate when 0.4–0.6, substantial when 0.6–0.8, and perfect when > 0.8. Ordinal weighting approach was applied for the following features: definition of non-enhancing and enhancing tumor margins, proportion of edema, midline crossing by edema/non-enhancing/enhancing tumor, calvarial remodeling, extent of resection for enhancing/non-enhancing tumor and edema, tumor size, presence of satellite lesions, diffusion characteristics, thickness of enhancing margin, T1/FLAIR signal ratio, tumor focality, proportions of necrosis, enhancement, non-enhancing tumor, and overall enhancement quality. Simple weighting was used for other features. We employed conditional confidence intervals to leverage specific data characteristics, providing more precise interval estimates which allows the generalizability of reproducibility findings to the larger rater (i.e., neuroradiologist) population. Statistical significance was set at a p-value < 0.05.

## Results

### Patients characteristics

A total number of 33 lesions were prospectively evaluated by three board-certified neuroradiologists independently. The mean age of patients was 47.8 years (95% confidence interval (CI) = 42.3 to 53.4). Of the included patients, 20 (60.6%) were male and 13 (39.4%) were female. The majority of the tumors, comprising 20 lesions, were glioblastoma, followed by astrocytoma (*n* = 7) and oligodendroglioma (*n* = 5). One tumor’s pathology was undefined (confirmed glioma pathology; however, the specific subtype was unknown). Moreover, 14 (42.4%) of tumors were right-sided, 15 (45.5%) were left-sided, and 4 (12.1%) were bilateral. Among the included tumors, 16 (48.4%) were located in the frontal lobe, 15 (45.4%) in the temporal lobe, 6 (18.1%) in the insular lobe, 6 (18.1%) in the parietal lobe, 3 (9.0%) in the occipital lobe, 2 (6.0%) in the brainstem, and 1 (3.0%) in the cerebellum. It is noteworthy that a single tumor may involve multiple regions of the brain.

### Agreement coefficients

**Fig. 3:**
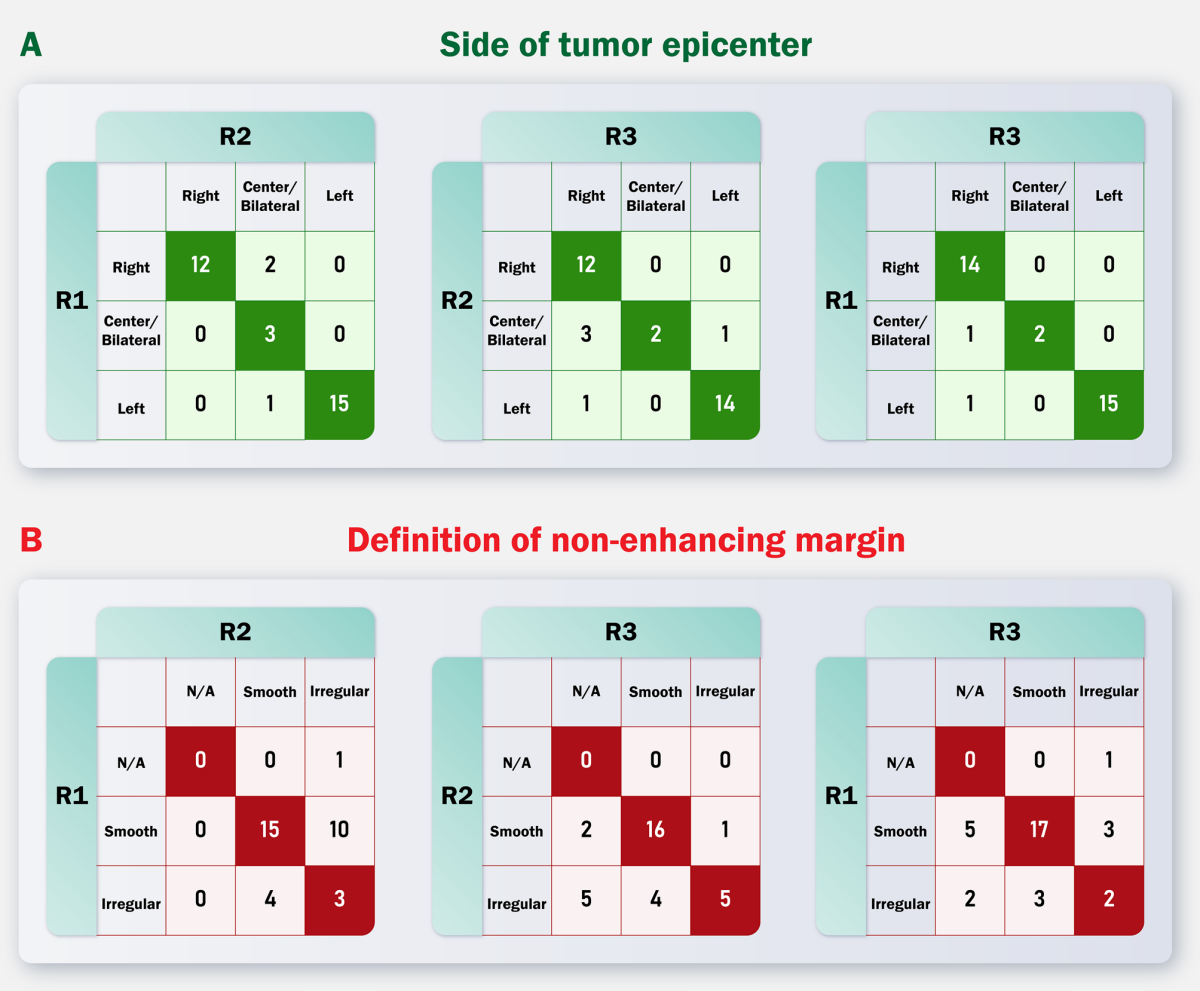
Pairwise comparison of each reader’s evaluation for the feature that had the best inter-reader agreement (side of tumor epicenter) and the feature that had the worst inter-reader agreement (definition of non-enhancing margin)

Most of the features showed good inter-rater reliability by the Gwet’s AC and Kappa analysis. Multifocality, enhancement quality, presence of cysts, enhanced tumor crossing of the midline, and presence of calvarial remodeling showed the highest level of agreement between features. On the other hand, diffusion characteristics, hemorrhage presence, involvement of eloquent brain regions, and T1/FLAIR ratio showed unacceptable reliability, with Gwet’s AC value of less than 0.6. This indicates that these features should not be used in defining clinical outcomes (e.g., diagnosis, prognosis, risk stratification, response to therapy, etc.) since different radiologists may report different and sometimes opposite imaging findings. In a number of features, the cut-off point of 0.6 was crossed by the Gwet’s AC confidence interval (i.e., borderline reproducibility), which are as follows: ependymal invasion, the definition of the non-enhancing tumor margin, the extent of enhancing tumor resection, tumor size, the proportion of edema, midline crossing by edema, deep white matter invasion, the proportion of nCET, the extent of nCET resection, the extent of vasogenic edema resection, pial invasion, midline crossing by nCET, cortical involvement, and thickness of the enhancing margin. Detailed results of agreement analyses for each feature are provided in Table 1. Figure 3 presents comparison of each reader’s evaluation for two example features.

**Table 1:** Agreement measures in tumor characteristics of patients ordered in Gwets kappa from highest to lowest

Feature	Gwet value	Gwet CI	Fleiss’ kappa	Fleiss’ kappa CI
Side of Tumor Epicenter	0.92	(0.87–0.97)	0.89	(0.82–0.96)
Edema Crosses Midline	0.87	(0.80–0.94)	0.80	(0.71–0.90)
Eloquent Brain	0.86	(0.80–0.92)	0.82	(0.75–0.89)
Enhancing tumor Crosses Midline	0.84	(0.75–0.92)	0.76	(0.65–0.87)
Enhancement Quality	0.84	(0.77–0.92)	0.78	(0.69–0.88)
Cyst(s)	0.84	(0.74–0.94)	0.76	(0.63–0.89)
Proportion Enhancing	0.83	(0.77–0.89)	0.79	(0.73–0.86)
nCET tumor Crosses Midline	0.82	(0.74–0.90)	0.75	(0.65–0.85)
Thickness of enhancing margin	0.81	(0.74–0.88)	0.76	(0.68–0.84)
Hemorrhage	0.81	(0.70–0.91)	0.70	(0.55–0.84)
Multifocal or Multicentric	0.80	(0.72–0.89)	0.68	(0.56–0.80)
Calvarial remodeling	0.80	(0.66–0.93)	0.66	(0.47–0.85)
Tumor size (longest perpendicular diameter)	0.80	(0.64–0.95)	0.63	(0.32–0.93)
Ependymal invasion	0.79	(0.68–0.89)	0.70	(0.58–0.83)
Proportion Necrosis	0.76	(0.69–0.82)	0.71	(0.64–0.78)
Extent of resection of enhancing tumor	0.76	(0.70–0.82)	0.71	(0.64–0.78)
Cortical involvement	0.75	(0.63–0.88)	0.64	(0.48–0.80)
Tumor size (longest diameter)	0.74	(0.67–0.80)	0.69	(0.61–0.76)
Proportion nCET	0.72	(0.66–0.79)	0.67	(0.59–0.74)
Proportion of Edema	0.72	(0.66–0.79)	0.67	(0.60–0.75)
Extent resection of nCET	0.70	(0.63–0.77)	0.64	(0.57–0.72)
Definition of the enhancing margin	0.69	(0.59–0.80)	0.59	(0.46–0.71)
Extent resection of vasogenic edema	0.68	(0.61–0.75)	0.62	(0.54–0.70)
Pial invasion	0.68	(0.57–0.80)	0.55	(0.41–0.70)
T1/FLAIR RATIO	0.67	(0.59–0.76)	0.59	(0.49–0.69)
Satellites	0.67	(0.55–0.80)	0.54	(0.39–0.69)
Diffusion	0.64	(0.55–0.73)	0.55	(0.45–0.65)
Deep WM invasion	0.62	(0.48–0.76)	0.47	(0.29–0.64)
Definition of the non-enhancing margin (e.g. Grade III)	0.61	(0.50–0.71)	0.48	(0.35–0.60)

CI: confidence interval, nCET: non-enhancing tumor

## Discussion

This study highlights the importance of standardized imaging assessments in glioma diagnosis and treatment planning by evaluating the inter-rater reliability of VASARI features. Our findings underscore the need for consistent reporting in clinical practice, particularly for features with poor agreement. The identification of reliable features, such as multifocality and enhancement quality, can inform the development of more robust clinical guidelines. Conversely, features with poor agreement, such as diffusion characteristics and T1/FLAIR ratio, necessitate further standardization to ensure consistent evaluations across different radiologists. The implications of our study are significant for enhancing diagnostic accuracy and patient outcomes. By focusing on features with high reliability, more clinically applicable tools may be developed, such as a modified version of VASARI or a new scoring system tailored to specific clinical requirements. This approach would promote broader implementation of these imaging features in standard clinical practice, thereby enhancing patient care in diverse clinical settings.

Our study showed that features like multifocality, enhancement quality, presence of cysts, enhanced tumor crossing of midline, presence of calvarial remodeling, proportion of enhancing tumor and necrosis, side of tumor epicenter, and presence of satellite lesions have good and robust reliability based on the Gwet’s AC and its narrow confidence interval. Previously several studies have been conducted to identify the prognostic predictive value of VASARI features, in addition to the development of prediction models to predict the recurrence of glioma and survival [12, 16–20] or in combination with clinical and genomic features, radiomics features, and machine learning approaches [21, 22]. Our results were in line with prior studies showing that enhancing tumor crossing the midline, multifocality, and ependymal invasion were relatively strong predictors of survival [11, 23]. Additionally, the results were similar to the results of another VASARI research project [24], which showed tumor side and tumor location, proportion of enhancing tumor, and presence of satellites to have the highest level of reliability. However, the diffusion characteristic was reported to have high agreement (generalized kappa = 0.730, 95% CI 0.664–0.828), whereas it showed unacceptable agreement in our study (kappa = 0.64, 95% CI = 0.55–0.73). Furthermore, the presence of calvarial remodeling showed low agreement (k = 0.36, 95% CI = 0.12–0.62) in contrast to our results (kappa = 0.80, 95% CI = 0.66–0.93).

Our study aimed to evaluate the inter-rater reliability of the 26 pre-operative VASARI features set in glioma imaging assessment. While the VASARI features were primarily designed for pre-surgical evaluations, we included three non-VASARI features, F26 (extent resection of enhancing tumors), F27 (extent resection of nCETs), and F28 (extent resection of vasogenic edema), which require both pre- and post-operative MRI scans to assess the extent of tumor resection and surrounding tissue characteristics. These features need post-operative MRI acquisition, so they may not be evaluated if this option is unavailable in clinical centers. Despite this, assessing these features from the routine clinical workup would result in the preserve of crucial post-operative information, as they inherently require a comparison between pre- and post-operative states to evaluate surgical outcomes. Our findings indicated moderate inter-reader agreement for these additional features. Moreover, poor agreement observed for certain features, such as diffusion characteristics, hemorrhage presence, involvement of eloquent brain regions, and T1/FLAIR ratio, can be attributed to several factors. Variability in image quality is a substantial contributor, as differences in MRI scanner technology, imaging protocols, and patient positioning can affect image clarity, leading to inconsistent interpretations of these features [25]. Additionally, differences in radiologists’ experience may influence how they assess certain features, with less experienced radiologists potentially struggling to consistently evaluate complex features without additional guidance The subjective nature of assessing these features also plays a crucial role [26]. Without clear operational definitions, radiologists may interpret features like diffusion characteristics and T1/FLAIR ratios differently, leading to poor agreement [26].

To address these challenges, dedicated training programs focusing on these features could improve inter-rater reliability. Furthermore, incorporating quantitative imaging techniques, such as dynamic susceptibility contrast perfusion-weighted imaging (DSC-PWI), may enhance the objectivity and reproducibility of glioma imaging assessments by providing more precise measurements of tumor characteristics [25]. Standardization and elaboration of these features are also crucial. Developing more detailed operational definitions or using clinical examples to illustrate the assessment of these features can help reduce ambiguity and subjectivity [27]. Conducting consensus meetings among radiologists to establish common criteria for evaluating these features can further improve consistency.

While our study primarily evaluated the inter-rater reliability of VASARI features among individual radiologists, the potential benefits of consensus readings in enhancing reliability warrant consideration. Consensus readings, where multiple radiologists agree on a diagnosis or feature assessment, can reduce variability and potentially improve diagnostic accuracy [14]. However, they may also introduce bias, particularly if less experienced radiologists are influenced by their more senior counterparts. This could lead to artificially inflated accuracy estimates in clinical studies [14]. Future studies could investigate whether consensus readings improve the reliability of VASARI features, particularly for those with poor agreement. This might involve comparing the inter-rater reliability of individual assessments versus consensus assessments for these features. Additionally, exploring the impact of consensus meetings on improving agreement among radiologists could provide valuable insights into enhancing the consistency of glioma imaging interpretations. The benefits of consensus readings include enhanced reliability through collaborative assessments, which can be particularly valuable for features with poor agreement, such as diffusion characteristics and T1/FLAIR ratios. However, the potential for bias and the time-consuming nature of consensus meetings are significant limitations. To mitigate these challenges, structured consensus protocols could be developed to ensure that all radiologists contribute equally to the decision-making process [14].

**Fig. 4:**
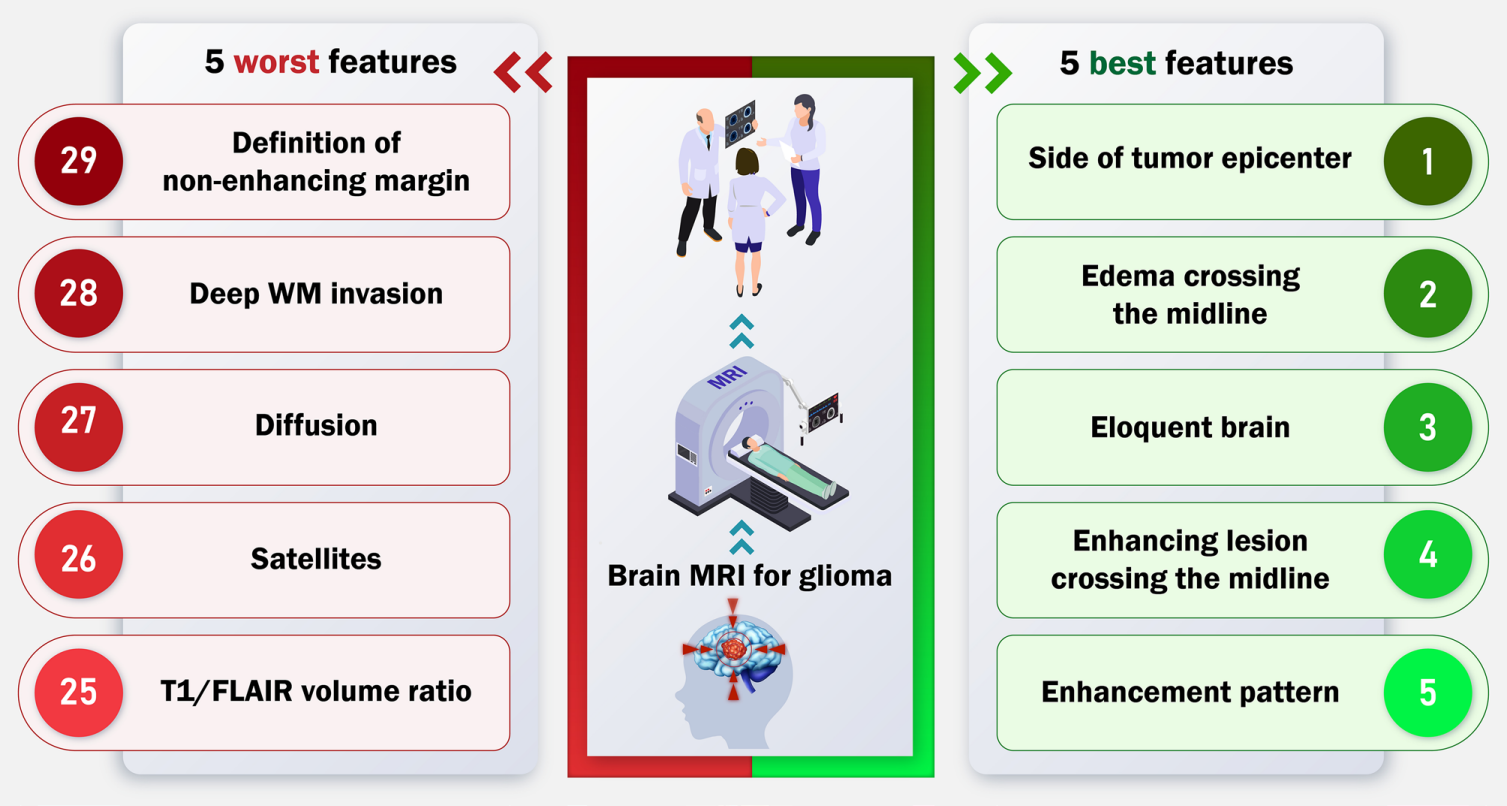
Graphical abstract containing key findings of the study

Features with high reliability could be incorporated into more clinically conductible tools like BT-RADS (Brain Tumors Reporting and Data System), as VASARI terminology is considered to be too time-consuming for clinical use [28]. By emphasizing features with higher inter-observer agreement, we can develop better scoring systems, such as a modified simpler version of VASARI or a new scoring system customized for particular clinical requirements. This would promote broader implementation of these imaging features in standard clinical practice, thereby enhancing patient care in different clinical settings. Additionally, the application of quantitative magnetic resonance techniques, such as dynamic susceptibility contrast perfusion-weighted imaging (DSC-PWI), has demonstrated promising results in distinguishing gliomas from other brain tumors [29, 30]. Integrating quantitative parameters, including cerebral blood volume and percentage of signal recovery, into the VASARI framework can potentially enhance the reproducibility and reliability of the imaging characteristics. This is primarily due to the objective nature of quantitative MR data, which may yield more consistent results compared to qualitative assessments performed by radiologists. Figure 4 illustrates a graphical abstract summarizing our findings in this study.

There were certain limitations pertaining to this study: (1) Some features of the VASARI set, despite being quantitative in nature, are categorized into different levels, which can limit the efficacy of the agreement studies. (2) The study involved three board-certified radiologists from a single center. Further studies using radiologists from different centers could also provide helpful. (3) The use of segmentation suggestions for readers in features such as the proportion of CET, nCET, and necrosis may have introduced an upgrading bias in our results.

## Conclusion

This study provides valuable insights into the reliability of VASARI features in glioma assessment. Radiologists showed strong agreement when assessing multifocality, enhancement quality, cyst presence, enhanced tumor crossing of midline, and calvarial remodeling. Conversely, assessments of diffusion characteristics, hemorrhage presence, involvement of eloquent brain regions, and T1/FLAIR ratio exhibited notably lower inter-rater agreement. These findings contribute to the ongoing refinement of standardized glioma reporting, potentially enhancing clinical decision-making and neuro-oncology research applications.

## Supplementary Information

Below is the link to the electronic supplementary material.

Supplementary Material 1

## Data Availability

Open access REMBRANDT dataset on https://www.cancerimagingarchive.net/collection/rembrandt/ was analyzed in this study.

## Declarations

### Animal study

N/A.

## References

[1] Wang LM, Englander ZK, Miller ML, Bruce JN. Malignant glioma. Adv Exp Med Biol. 2023;1405:1–30. 37452933 10.1007/978-3-031-23705-8_1

[2] Ostrom QT, Gittleman H, Truitt G, Boscia A, Kruchko C, Barnholtz-Sloan JS. CBTRUS statistical report: primary brain and other central nervous system tumors diagnosed in the United States in 2011–2015. Neuro Oncol. 2018;20(suppl4):iv1–86. 30445539 10.1093/neuonc/noy131 PMC6129949

[3] Falk Delgado A. Advances of MR imaging in glioma: what the neurosurgeon needs to know. Acta Neurochir. 2025;167(1):174. 40542873 10.1007/s00701-025-06593-6 PMC12182469

[4] Hu YC, Yan LF, Sun Q, Liu ZC, Wang SM, Han Y, et al. Comparison between ultra-high and conventional mono b-value DWI for preoperative glioma grading. Oncotarget. 2017;8(23):37884–95. 28039453 10.18632/oncotarget.14180 PMC5514959

[5] Parvez K, Parvez A, Zadeh G. The diagnosis and treatment of pseudoprogression, radiation necrosis and brain tumor recurrence. Int J Mol Sci. 2014;15(7):11832–46. 24995696 10.3390/ijms150711832 PMC4139817

[6] Almalki YE, Basha MAA, Metwally MI, Zeed NA, Nada MG, Alduraibi SK et al. Validating brain tumor reporting and data system (BT-RADS) as a diagnostic tool for glioma follow-up after surgery. Biomedicines. 2024;12(4). 10.3390/biomedicines12040887 PMC11048183 38672241

[7] Pons-Escoda A, Majos C, Smits M, Oleaga L. Presurgical diagnosis of diffuse gliomas in adults: Post-WHO 2021 practical perspectives from radiologists in neuro-oncology units. Radiologia (Engl Ed). 2024;66(3):260–77. 38908887 10.1016/j.rxeng.2024.03.002

[8] Wegscheid ML, Jennings JW. VASARI glioma features: insights into their impact and performance. Radiol Imaging Cancer. 2024;6(5):e249018. 39269896 10.1148/rycan.249018 PMC11443470

[9] Smits M. MRI biomarkers in neuro-oncology. Nat Reviews Neurol. 2021;17:1–15. 10.1038/s41582-021-00510-y 34149051

[10] Thust SC, Heiland S, Falini A, Jäger HR, Waldman AD, Sundgren PC, et al. Glioma imaging in europe: A survey of 220 centres and recommendations for best clinical practice. Eur Radiol. 2018;28(8):3306–17. 29536240 10.1007/s00330-018-5314-5 PMC6028837

[11] Wang L, Wei L, Wang J, Li N, Gao Y, Ma H, et al. Evaluation of perfusion MRI value for tumor progression assessment after glioma radiotherapy: A systematic review and meta-analysis. Med (Baltim). 2020;99(52):e23766. 10.1097/MD.0000000000023766 PMC7769293 33350761

[12] Azizova A, Prysiazhniuk Y, Wamelink I, Petr J, Barkhof F, Keil VC. Ten years of VASARI glioma features: systematic review and meta-analysis of their impact and performance. AJNR Am J Neuroradiol. 2024;45(8):1053–62. 38937115 10.3174/ajnr.A8274 PMC11383402

[13] Scarpace L, Flanders A, Jain R, Mikkelsen T, Andrews D. Data from REMBRANDT [Data set]. The Cancer Imaging Archive. 2019.

[14] Gwet K. Handbook of inter-rater reliability: the definitive guide to measuring the extent of agreement among raters. 2012.

[15] Sayah A, Bencheqroun C, Bhuvaneshwar K, Belouali A, Bakas S, Sako C, et al. Enhancing the REMBRANDT MRI collection with expert segmentation labels and quantitative radiomic features. Sci Data. 2022;9(1):338. 35701399 10.1038/s41597-022-01415-1 PMC9198015

[16] Schmainda KM, Prah MA, Hu LS, Quarles CC, Semmineh N, Rand SD, et al. Moving toward a consensus DSC-MRI protocol: validation of a low–flip angle single-dose option as a reference standard for brain tumors. Am J Neuroradiol. 2019;40(4):626–33. 30923088 10.3174/ajnr.A6015 PMC6461489

[17] Sacli-Bilmez B, Firat Z, Topcuoglu OM, Yaltirik K, Ture U, Ozturk-Isik E. Identifying overall survival in 98 glioblastomas using VASARI features at 3T. Clin Imaging. 2023;93:86–92. 36417792 10.1016/j.clinimag.2022.10.011

[18] Han Y, Wang YY, Yang Y, Qiao SQ, Liu ZC, Cui GB, et al. Association between dichotomized VASARI feature and overall survival in glioblastoma patients: a single-institution propensity score matching analysis. Cancer Imaging: Official Publication Int Cancer Imaging Soc. 2024;24(1):109. 10.1186/s40644-024-00754-z PMC11330608 39155364

[19] Rios Velazquez E, Meier R, Dunn WD Jr., Alexander B, Wiest R, Bauer S, et al. Fully automatic GBM segmentation in the TCGA-GBM dataset: prognosis and correlation with VASARI features. Sci Rep. 2015;5:16822. 26576732 10.1038/srep16822 PMC4649540

[20] Peeken JC, Hesse J, Haller B, Kessel KA, Nüsslin F, Combs SE. Semantic imaging features predict disease progression and survival in glioblastoma multiforme patients. Strahlentherapie und Onkologie: Organ der Deutschen Rontgengesellschaft. 2018;194(6):580–90. 10.1007/s00066-018-1276-4 29442128

[21] van Dijken BR, van Laar PJ, Holtman GA, van der Hoorn A. Diagnostic accuracy of magnetic resonance imaging techniques for treatment response evaluation in patients with high-grade glioma, a systematic review and meta-analysis. Eur Radiol. 2017;27:4129–44. 28332014 10.1007/s00330-017-4789-9 PMC5579204

[22] Lu Y, Patel M, Natarajan K, Ughratdar I, Sanghera P, Jena R, et al. Machine learning-based radiomic, clinical and semantic feature analysis for predicting overall survival and MGMT promoter methylation status in patients with glioblastoma. Magn Reson Imaging. 2020;74:161–70. 32980505 10.1016/j.mri.2020.09.017

[23] Yeniçeri İÖ, Yıldız ME, Özduman K, Danyeli AE, Pamir MN, Dinçer A. The reliability and interobserver reproducibility of T2/FLAIR mismatch in the diagnosis of IDH-mutant Astrocytomas. Diagn Interventional Radiol. 2021;27(6):796. 10.5152/dir.2021.20624 PMC8621633 34792037

[24] Ebaid NY, Ahmed RN, Assy MM, Amin MI, Eldin AMA, Alsowey AM, et al. Diagnostic validity and reliability of BT-RADS in the management of recurrent high-grade glioma. J Neuroradiol. 2024;51(4):101190. 38492800 10.1016/j.neurad.2024.03.001

[25] Sanvito F, Kaufmann TJ, Cloughesy TF, Wen PY, Ellingson BM. Standardized brain tumor imaging protocols for clinical trials: current recommendations and tips for integration. Front Radiol. 2023;3:1267615. 38152383 10.3389/fradi.2023.1267615 PMC10751345

[26] Due-Tønnessen P, Pinho MC, Emblem KE, Hald JK, Kanoto M, Abildgaard A, et al. The impact of MRI features and observer confidence on the treatment decision-making for patients with untreated glioma. Sci Rep. 2019;9(1):19898. 31882644 10.1038/s41598-019-56333-x PMC6934740

[27] Setyawan NH, Choridah L, Nugroho HA, Malueka RG, Dwianingsih EK. Beyond invasive biopsies: using VASARI MRI features to predict grade and molecular parameters in gliomas. Cancer Imaging: Official Publication Int Cancer Imaging Soc. 2024;24(1):3. 10.1186/s40644-023-00638-8 PMC10759759 38167551

[28] Wegscheid ML, Jennings JW. VASARI glioma features: insights into their impact and performance. Radiol: Imaging Cancer. 2024;6(5):e249018. 10.1148/rycan.249018 PMC11443470 39269896

[29] Pons-Escoda A, Garcia-Ruiz A, Naval-Baudin P, Martinez-Zalacain I, Castell J, Camins A, et al. Differentiating IDH-mutant Astrocytomas and 1p19q-codeleted oligodendrogliomas using DSC-PWI: high performance through cerebral blood volume and percentage of signal recovery percentiles. Eur Radiol. 2024;34(8):5320–30. 38282078 10.1007/s00330-024-10611-z PMC11255054

[30] Pons-Escoda A, Naval-Baudin P, Viveros M, Flores-Casaperalta S, Martinez-Zalacaín I, Plans G, et al. DSC-PWI presurgical differentiation of grade 4 Astrocytoma and glioblastoma in young adults: rCBV percentile analysis across enhancing and non-enhancing regions. Neuroradiology. 2024;66(8):1267–77. 38834877 10.1007/s00234-024-03385-0 PMC11246293

