# Supplementary material for: Assessing inter-rater reliability of MRI features in glioma: a multi-radiologist agreement study
Source: BMC Med Imaging. 2025 Nov 20;25:480. doi: 10.1186/s12880-025-01941-5 (PMC12636226; doi:10.1186/s12880-025-01941-5)
Supplement: Supplementary file 1 — Supplementary Material 1 [file 12880_2025_1941_MOESM1_ESM.docx]

Analyzing the agreement among radiologists on the preoperative MRI characteristics of patients with glioma

1. Review question:

To compare the level of agreement among radiologists regarding the characteristics of preoperative MRI in patients with glioma

1. Condition or domain being studied:

Gliomas are the most common type of primary brain tumors that develop from glial or progenitor cells. They include astrocytoma (including glioblastoma), oligodendroglioma, ependymoma, oligoastrocytoma (mixed glioma), malignant glioma, not otherwise specified (NOS), and a small number of rare histologies. Glioblastoma, commonly referred to as Grade IV glioma, makes up 56% of gliomas in adults and is linked to a notably unfavorable prognosis, with a median survival of 18 months.

Magnetic resonance imaging (MRI) is the primary imaging technique used to evaluate brain tumors before and after surgery. It plays a crucial role in optimizing the treatment of brain tumors. However, it can be difficult to interpret brain tumor MRI findings because the changes seen on MR imaging are frequently not a result of increased tumor activity but rather a reaction to the treatment. The existence of edema, inflammation, or heightened permeability of the vascular or blood-brain barrier makes it difficult to distinguish between real tumor growth and the reaction to treatment. Various established criteria have been proposed to assess the response in gliomas, such as the World Health Organization, Levin, MacDonald, Response Assessment in Neuro-Oncology (RANO), and Brain Tumor Reporting and Data System (BT-RADS). However, the adoption of these applications in radiology has been limited for various reasons, including the need for external validation, substantial variation in interpretation among different observers, and their inherent complexity, which can lead to confusion among physicians.

1. Participants/population:

We will use a dataset consisting of 33 patients who were diagnosed with glioma and recruited from the Thomas Jefferson University (TJU) dataset. The data was collected from the institutional database. Patients' available consent was secured for this study due to open access and accessible data availability.

1. Main outcome:

To assess the inter-rater agreement among radiologists about the specific features of MRI scans in patients diagnosed with glioma and then rank the features based on kappa/Gwen coefficients of agreement.

1. Imaging:

The pre- and post-procedural studies were evaluated by three board-certified radiologists. The researchers conducted separate measurements of tumors in both pre- and post-procedural examinations, including factors such as tumor location, enhancement quality, proportion enhancement, proportion necrosis, and thickness of the enhancing margin.

1. Strategy for data synthesis:

The analysis will be conducted using STATA version 17.0 and MedCalc 22.0.

1. Contact details for further information:

Afshin Mohammadi

mohammadi.a@umsu.ac.ir

1. Team members:

Alisa Mohebbi

Saeed Mohammadzadeh

Ali Abdi

1. Type and method of research:

Original study evaluating agreement

1. Anticipated or actual start date:

June 31, 2024

1. Anticipated completion date:

July 20, 2024

1. Funding sources/sponsors:

None

1. Conflicts of interest:

None
